# Condom use and alcohol consumption in adolescents and youth

**DOI:** 10.1590/S1679-45082016AO3677

**Published:** 2016

**Authors:** Rachel Mola, Ana Carolina Rodarti Pitangui, Sháyra Anny Moura Barbosa, Layane Sá Almeida, Mayara Ruth Marinho de Sousa, Wellypâmela Pauliny de Lima Pio, Rodrigo Cappato de Araújo

**Affiliations:** 1 Universidade de Pernambuco, Petrolina, PE, Brazil.

**Keywords:** Risk-taking, Alcoholism, Binge drinking, Contraception, Sexual behavior

## Abstract

**Objective:**

To determine the association between not using the male condom and alcohol consumption in adolescents and schoolchildren.

**Methods:**

An epidemiological study, with a cross-sectional, descriptive, and correlation design carried out from March to July 2014. The sample consisted of students in public primary and secondary education, aged between 12 and 24 years. The social and demographic survey and the Youth Risk Behavior Survey questionnaire were used.

**Results:**

The study included 1,275 students, of these; 37.0% reported having had sexual relations. The prevalent age of sexual initiation was 14-16 years 55.7% and 65.6% used condom in the last sexual intercourse. Regarding the lack of condom use at the last intercourse, girls showed an association with drunkenness in the previous 30 days (2.19; 95%CI: 1.06-4.54).

**Conclusion:**

In females, the non-use of condoms was associated with drunkenness in the previous 30 days.

## RESUMO

**Objetivo:**

Identificar os fatores associados ao não uso de preservativo masculino e ao consumo de bebida alcoólica em adolescentes e jovens escolares.

**Métodos:**

Estudo epidemiológico, com delineamento transversal, descritivo e correlacional, desenvolvido de março a julho de 2014. A amostra foi composta por estudantes dos Ensinos Fundamental e Médio da rede pública estadual, com idades entre 12 e 24 anos. Empregaram-se o inquérito sociodemográfico e o questionário *Youth Risk Behavior Survey*.

**Resultados:**

Foram incluídos 1.275 estudantes; 37,0% deles relataram terem tido relação sexual. A idade prevalente de iniciação sexual foi de 14 a 16 anos, com 55,7%; 65,6% usaram preservativo na última relação. Com relação ao não uso de preservativo na última relação, as meninas apresentaram associação com bebedeira nos últimos 30 dias (2,19; IC95%: 1,06-4,54).

**Conclusão:**

O não uso de preservativos esteve associado com bebedeira nos últimos 30 dias nas meninas.

## INTRODUCTION

Risky sexual behavior is pointed out as a consequence of unprotected sex.^(1)^The male condom is the most well-known contraceptive method among adolescents,^(2)^and has shown, over the last years, greater distribution and an increase in its use as a result of different forms of intervention directed at this population.^(3)^

Nevertheless, its use is still variable along the course of the amorous and sexual life of each individual.^(3)^ Despite the fact that many adolescents report its use, a large portion of this population still exhibits a behavior that causes concerns.^(4)^ Data from the National Health Survey of Students *[Pesquisa Nacional de Saúde do Escolar]* (PeNSE) reveals that one in every five adolescents states not having used a male condom during their last sexual intercourse. Therefore they are more vulnerable to the risk of sexually transmitted diseases (STD), AIDS, and/or early pregnancy/parenthood.^(4)^

Yet, the use of the male contraceptive among Brazilian adolescents is still more common among boys than girls during their first intercourse,^(5)^ a situation that is repeated when the latest sexual encounter is analyzed.^(6)^ This unsafe behavior is the most prevalent condition among the girls and the students who attend public schools. Some factors, such as advanced age, low socioeconomic condition,^(6)^not receiving information on sexual and reproductive health at school,^(4)^ and low level of maternal schooling are associated with unprotected sex.^(7)^

The adoption of unhealthy habits, such as unprotected sex and the excessive consumption of alcohol, academic problems, substance abuse, and delinquent behavior are associated with the initiation at an early age of alcoholic beverages.^(8,9)^ The consumption of alcohol has been associated as a means to reach pleasure, beauty, financial and sexual success. In face of the image sold by advertising, adolescents believe the alcoholic beverage commercials to be true, and seek similarity in situations in their own lives, characterizing a significant risk factor for its abusive consumption.^(10)^In this way, currently, the incentive and permissiveness as to alcohol consumption is made evident by the advertising market.^(11)^

In Brazil, the studies that cover the use of the male condom and the consumption of alcohol are mostly carried out with samples from large cities, and this theme is rarely studied with a focus on the population from the interior of the country. Additionally, there are numerous negative repercussions as to what these behaviors can cause in the lives of the adolescents and youth. Thus, it is believed that information that covers other regions might contribute towards the identification of risk groups and patterns, enabling monitoring of the health levels of adolescents and youth with the purpose of creating targeted programs and policies of health promotion.

## OBJECTIVE

To identify the factors associated with the nonuse of the male condom and to the consumption of alcoholic beverages in school-aged adolescents and young person.

## METHODS

This is a school-based epidemiological study, with a cross-sectional, descriptive, and correlational design, which was conducted in state-owned Primary and Secondary Education schools, situated in the city of Petrolina (PE), during the period from March to July, 2014. The population studied was made up of students - adolescents (12 to 19 years) and youth (15 to 24 years). A population of 25,635 students of public Primary and Secondary Education of Petrolina (PE) was considered.

The distribution of schools was made according to their size to guarantee proportional samples. Thus, they were classified into three size categories: small (less than 200 students), medium (200 to 499 students), and large (500 students or more). The students enrolled in the morning and afternoon periods were grouped into a single category (daytime students). For the sample selection, simple random sampling was used by conglomerates in two stages, in which “school” and the “group” represented the sample units, respectively, for the first and second phases. All 29 urban state-owned schools in Petrolina (PE) were considered eligible for inclusion in the study. Initially, a drawing was done of the schools according to size. School randomization was performed using the WINPEPI software. After all steps, nine Primary and Secondary Education schools were selected, which represented 31.03% of the state school system of the city of Petrolina (PE).

For quantification of the minimum sample, the WINPEPI software was used, considering a population of 25,635 students, with a 95%CI, a maximal tolerable error of 4%, and sample loss of 20%, and since it was a study covering the analysis of different risk behaviors (nonuse of the male condom and alcohol consumption) with varied frequencies of occurrence, the estimated prevalence used was 50%, totaling the quantity of 474 adolescents. Multiplication of the minimal size of the sample was made by 2.0 (sample outline effect), totaling 948 adolescents, but the final sample was made up of 1,326 adolescents. All students of the groups selected that satisfied the criteria for inclusion were invited to participate in the study.

The inclusion criteria were be characterized as an adolescent or youth, as per the definition of the World Health Organization (WHO) from both genders; know how to read and write in Portuguese; be duly enrolled in an institution located in the urban zone of the city of Petrolina; and not present with neurological pathologies or alterations in physical condition that would impede the completion of the data collection instrument. The study excluded adolescents that did not inform their gender or age on the questionnaire, as well as those that did not correctly fill in the answers to the questions.

The term “drunkenness” was defined as the ingestion of five or more doses of alcoholic beverage on one occasion.^(6,12,13)^ The type of alcohol beverage user was classified in the following manner: nonuser (has not drunk an alcoholic beverage at any moment in his/her life); new user/experimentation (if the person drank one to 19 days in his/her life); moderate user (if the person drank on 20 to 99 days), and heavy user (100 or more days of ingestion of an alcoholic beverage in his/her life).^(13)^

### Data collection instruments

For data collection, two instruments were used: 1) the socioeconomic survey, established by means of a self-applied structured questionnaire based on the criteria of the Brazilian Institute of Geography and Statistics (IBGE), which had questions related to social and demographic (marital status, religion, having children, level of schooling of the mother and father) and economic aspects (monthly family income in terms of minimum wages) nature; and 2) the questionnaire on risk behaviors Youth Risk Behavior Survey (YRBS), comprising 87 questions covering six categories that contribute towards triggering morbidities, mortality, and social problems among young people. Various studies have demonstrated that this is a valid and reliable instrument,^(14)^ and the validation of the Brazilian version was made by Guedes et al.^(15)^

In this study, the domains related to the consumption of alcoholic beverages and sexual behavior, composed by questions 38 to 43, and 57 to 64, respectively, were used for the analysis. The values of the Kappa agreement index for the two domains varied from moderate to substantial, with values between 49.4 and 66.7 for consumption of alcoholic beverages, and excellent, with values between 81 and 95.6 for sexual behavior.^(15)^

### Collection procedures

Prior to the data collection, a pilot study was done to determine possible biases and limitations in the research procedures. This step served to measure the time of application of the instrument and training of researchers. The data were collected at a state-owned Primary and Secondary Education school of Petrolina (PE), with a sample of 80 adolescents. Ten duly trained researchers participated.

The investigation began with the presentation of the agreement letter from the State Department of Education of Pernambuco to the headmasters of each selected school. The presentation and familiarization with the project initially occurred by means of announcements in the selected schools. The Informed Consent Form (ICF) and the Agreement Form were given to the students, so that they could take this information about the investigation to their parents or guardians. Next, data collection began, and the volunteers were submitted to the socioeconomic inquiry and questioning about risk behaviors.

The volunteers were organized in the classroom and invited to participate in the study, receiving an anonymous self-explanatory questionnaire that was completed in the absence of the teacher. These procedures were done so that there was no interference from the teachers, thus minimizing possible inducement or embarrassment when filling out the instruments.

After the questionnaire was completed, the students were instructed to hand in the instrument to the investigator. Finally, the researchers deposited the questionnaires, face down, on a table at the front of the room, and the teens were invited to leave the classroom. The volunteers were continually assisted by the researchers, who were instructed to clarify any possible doubts as to filling in the information, and not interfering with the answers. The questionnaire took, on average, 40 minutes to be completed.

All participants received explanations as to the proposed objectives and methodologies by means of the ICF. The adolescents and at least one of the parents or legal guardians (if the individual was underage) that agreed to participate in the study, signed the Agreement Form and the ICF, respectively.

The study was approved by the Research Ethics Committee of the University of Pernambuco, Official Opinion number 521.340, CAAE: 24288213.2.0000.5207, and adhered to the precepts of Resolution 466/12 of the National Health Council and of the Statute of the Child and Adolescent.

### Statistical analysis

The data were recorded by double entry on Microsoft Excel software and processed and analyzed using the Statistical Package for the Social Sciences (SPSS), version 20.0. Initially, the normality of the data was verified by means of the Kolmogorov-Smirnov test. In the case of symmetrical distribution, the central tendency measure and dispersion was used for presentation of the continuous variables, or measurement of the central tendency added to separatrices, for non-parametric distribution. Categorical data were presented in absolute and relative frequencies, and possible associations were calculated by the χ^2^ test. The *odds ratio* was calculated with a 95%CI. By means of a previously established model of chances, independent variables were selected that presented with a level of significance of 0.20 for the analysis of a binary logistic multiple regression model, adjusted for gender, after analysis of the possible confounding and interaction factors. In this model, the insert (Enter) method was used. The Omnibus and Hosmer-Lemeshow tests were used to describe the validity and the explanatory power of the final model, respectively. All tests were two-tailed, and in all analyses, a p<0.05 significance level was adopted.

## RESULTS

Of the 1,326 students evaluated, 1,275 were included in the final data analysis. Fifty-one individuals were excluded for not completing relevant information for the analysis. The greatest portion of the sample was composed of girls, aged between 14 and 16 years, mulatto, Catholic, single, whose parents had completed Primary school or had not completed Secondary school, and had a low family income. Due to the inclusion criteria, a smaller portion was in the seventh year [of schooling], and the distribution was similar among the other school years. Table 1 characterizes the social-demographic and economic profiles of the adolescents and youth evaluated.

**Table 1:** Distribution of the social-demographic and economic characteristics of adolescents and youth

Variables	Female	Male	Total
	n (%); 95%CI	n (%); 95%CI	n (%); 95%CI
Age			
12-13	177 (24.8); 21.6-28.0	135 (24.2); 20.7-28.0	312 (24.5); 22.2-27.0
14-16	382 (53.4); 49.7-57.1	279 (50.1); 45.8-54.3	661 (52.0); 49.2-54.7
≥17	156 (21.8); 18.8-25.0	143 (25.7); 22.0-29.5	299 (23.5); 21.2-25.9
Skin color			
White	143 (20.1); 17.2-23.3	116 (20.9); 17.5-24.4	256 (20.5); 18.1-22.6
Black	79 (11.1); 8.9-13.6	95 (17.1); 14.0-20.4	174 (13.7); 11.9-15.8
Mulatto	409 (57.6); 53.8-61.2	288 (51.8); 47.5-56.0	697 (55.1); 52.4-57.9
Other	79 (11.1); 8.9-13.6	57 (10.3); 7.8-13.0	136 (10.7); 9.1-12.6
Religion			
Catholic	385 (54.4); 50.6-58.0	245 (45); 40.8-49.3	630 (50.3); 47.5-53.1
Evangelical	193 (27.3); 24.0-30.7	162 (29.8); 25.9-33.8	355 (28.4); 25.8-30.9
Other	23 (3.2); 2.0-4.8	25 (4.6); 3.0-6.7	48 (3.8); 2.8-5.0
None	107 (15.1); 12.5-17.9	112 (20.6); 17.2-24.2	219 (17.5); 15.4-19.2
Marital status			
Single	670 (94.5); 93.0-96.1	515 (94.7); 92.4-96.4	1.185 (94.6); 93.2-95.8
Married	38 (5.4); 3.8-7.3	22 (4.0); 2.5-6.1	60 (4.8); 3.7-6.1
Other	1 (0.1); 0.0-0.8	7 (1.3); 0.5-2.6	8 (0.6); 0.3-1.2
Grade/level			
7^th^/Primary Education	59 (8.3); 6.4-10.6	65 (11.7); 9.3-15.0	124 (9.8); 8.2-11.5
8^th^/Primary Education	137 (19.2); 16.5-22.3	118 (21.3); 18.3-25.3	255 (20.1); 17.9-22.4
9^th^/Primary Education	105 (14.7); 12.2-17.6	106 (19.1); 16.2-23.1	211 (16.6); 14.6-18.8
1^st^/Secondary Education	146 (20.4); 17.7-23.8	81 (14.6); 11.3-19.1	227 (17.9); 15.8-20.1
2^nd^/Secondary Education	134 (18.7); 16.1-22.0	84 (15.2); 12.5-18.8	218 (17.2); 15.1-19.4
3^rd^/Secondary Education	134 (18.7); 16.1-22.0	100 (18.1); 15.2-21.9	234 (18.4); 16.3-20.7
Father’s schooling level			
Illiterate or incomplete Primary Education	160 (33.1); 28.9-37.5	112 (28.4); 23.9-33.1	272 (31.0); 27.9-34.2
Complete Primary Education or incomplete Secondary Education	157 (32.5); 28.3-36.8	145 (36.7); 31.9-41.7	302 (34.4); 31.2-37.6
Complete Secondary Education or incomplete Further Education	108 (22.4); 17.7-27.5	80 (20.3); 16.4-24.6	188 (21.4); 18.7-24.3
Complete Further Education	58 (12.0); 9.2-15.2	58 (14.7); 11.3-18.6	116 (13.2); 11.0-15.6
Father’s schooling level			
Illiterate or incomplete Primary Education	122 (21.9); 18.5-25.5	78 (18.1); 14.6-22.1	200 (20.2); 17.7-22.8
Complete Primary Education or incomplete Secondary Education	184 (33.0); 29.1-37.0	144 (33.4); 29.0-38.1	328 (33.2); 30.2-36.2
Complete Secondary Education or incomplete Further Education	168 (30.1); 26.3-34.1	131 (30.4); 26.1-35.0	299 (30.2); 27.3-33.2
Complete Further Education	84 (15.1); 12.2-18.3	78 (18.1); 14.6-22.1	162 (16.4); 14.1-18.8
Monthly family income*			
1-2	304 (70.5); 66.0-75.0	194 (59.7); 54.1-65.1	498 (65.9); 62.3-69.2
3-5	97 (22.5); 18.7-26.8	103 (31.7); 26.7-37.1	200 (26.5); 23.3-29.7
>5	30 (7.0); 4.8-9.8	28 (8.6); 5.8-12.2	58 (7.7); 5.8-9.8

The total number may differ due to lost values. *Monthly family income based on minimum wages estimated at a value of R$ 724.00. 95%CI: 95% confidence interval.

As to the sexual behavior, 461 (37.0%) students reported already having had sexual intercourse before the time of the survey, and most of them had started their sexual life before the age of 17 years. A high percentage reported not having consumed alcoholic beverages and not having used the male condom in their latest sexual encounter, and this is the most frequently used method. On table 2, one can visualize the distribution of sexual behavior of the adolescents and youth that reported having an active sexual life.

**Table 2:** Distribution of sexual behavior of adolescents and youth who reported active sexual life

Variables	Female	Male	Total
	n (%); 95%CI	n (%); 95%CI	n (%); 95%CI
Age of sexual initiation			
≤13	45 (21.7); 16.3-27.9	133 (50.6); 44.3-56.7	178 (37.9); 33.4-42.4
14-16	142 (68.6); 61.8-74.8	120 (45.6); 39.5-51.8	262 (55.7); 51.1-60.2
≥17	20 (9.7); 6.0-14.5	10 (3.8); 1.8-6.8	30 (6.4); 4.3-8.9
Consumption of alcoholic beverage in the last intercourse			
Yes	22 (10.2); 6.5-15.0	37 (14.2); 10.2-19.0	59 (12.4); 9.5-15.7
No	193 (89.8); 84.9-93.4	223 (85.8); 80.9-89.7	416 (87.6); 84.2-90.4
Use of male condom in the last intercourse			
Yes	127 (62.0); 54.9-68.6	184 (68.4); 62.4-73.9	311 (65.6); 61.1-69.8
No	78 (38.0); 31.3-45.0	85 (31.6); 26.0-37.5	163 (34.4); 30.1-38.8
Contraceptive method used in the last sexual intercourse			
None	31 (15.2); 10.5-20.8	49 (18.8); 14.3-24.1	80 (17.2); 13.9-21.0
Oral contraceptive	32 (15.7); 10.9-21.4	20 (7.7); 4.8-11.6	52 (11.2); 8.5-14.4
Male condom	92 (45.1); 38.1-52.2	155 (59.6); 53.4-65.6	247 (53.2); 48.6-57.9
Injectable contraceptive	15 (7.4); 4.1-11.8	2 (0.8); 0.0-2.8	17 (3.7); 2.2-5.8
*Coitus interruptus*	10 (4.9); 2.3-8.8	15 (5.8); 3.2-9.3	25 (5.4); 3.5-7.8
Another oral	2 (1.0); 0.1-3.5	5 (1.9); 0.6-4.4	7 (1.5); 0.6-3.1
Contraceptive + male condom	22 (10.8); 6.9-15.9	14 (5.4); 2.9-8.9	36 (7.8); 5.5-10.6

The total number may differ due to lost values. 95%CI: 95% confidence interval.

As to the profile of adolescents and youth related to the consumption of alcoholic beverages, table 3 displays the distribution of the variables analyzed. Despite the majority of the students having begun using alcoholic beverages early in life, they still were classified as nonusers or new users, with a low percentage of ingestion of doses and of involvement with drunkenness in the previous 30 days.

**Table 3:** Distribution of alcoholic beverage among adolescents and youth

Variables	Female	Male	Total
	n (%); 95%CI	n (%); 95%CI	n (%); 95%CI
Age at first dose			
≤12	147 (36.1); 31.4-41.0	136 (44.0); 38.4-49.7	283 (39.5); 35.9-43.2
13-14	134 (32.9); 28.4-37.7	83 (26.9); 22.0-32.2	217 (30.3); 26.9-33.8
≥15	126 (31.0); 26.5-35.7	90 (29.1); 24.1-34.5	216 (30.2); 26.8-33.7
Days that have drunk in life			
Non-user	333 (46.9); 43.2-50.7	267 (47.9); 43.7-52.2	600 (47.4); 44.6-50.2
New user/experimenting	260 (36.6); 33.1-40.3	190 (34.1); 30.2-38.2	450 (35.5); 32.9-38.2
Moderate user	83 (11.7); 9.4-14.3	59 (10.6); 8.1-13.5	142 (11.2); 9.5-13.1
Heavy user	34 (4.8); 3.3-6.6	41(7.4); 5.3-9.9	75 (5.9); 4.7-7.4
Doses that has drunk in the last 30 days			
0	582 (81.9); 78.8-84.6	404 (72.8); 68.9-76.5	918 (72.5); 69.9-74.9
1-9	116 (16.3); 13.7-19.2	133 (24); 20.5-27.7	314 (24.8); 22.4-27.3
10-29	16 (2.3); 1.3-3.6	14 (2.5); 1.4-4.2	30 (2.4); 1.6-3.4
≥30	-	4 (0.7); 0.2-1.8	4 (0.3); 0.1-0.8
Drunkenness in the last 30 days			
0	582 (81.9); 78.8-84.6	442 (79.5); 75.9-82.8	1,024 (80.8); 78.5-82.9
1-5	116 (16.3); 13.7-19.2	100 (18.0); 14.9-21.4	216 (17.0); 15.0-19.2
6-19	8 (1.1); 0.4-2.2	8 (1.4); 0.6-2.8	16 (1.3); 0.7-2.0
≥20	5 (0.7); 0.2-1.6	6 (1.1); 0.4-2.3	11 (0.9); 0.4-1.5

The total number may differ due to lost values. 95%CI: 95% confidence interval.

Table 4 shows the analysis related to the use of male condom and its association with the independent variables studied among the adolescents and youth. Although there was no statistically significant difference in the associations between the use of male condom and the variables analyzed, gender, involvement in drunkenness, schooling level, mother’s schooling level, and the use of alcoholic beverages at some time in life showed values to be inserted into the binary regression model.

**Table 4:** Association between the use of male condom with the independent variables studied in adolescents and youth

Independent variables	Use of male condom			

	Yes	No	Total	p value
	n (%)	n (%)	n (%)	
Age				
≤14	49 (15.9)	33 (20.2)	82 (17.4)	0.285
≥15	260 (84.1)	130 (79.8)	390 (82.6)	
Gender				
Female	127 (40.8)	78 (47.9)	205 (43.2)	0.172^*^
Male	184 (59.2)	85 (52.1)	269 (56.8)	
Religion				
Yes	70 (23.4)	33 (20.4)	103 (22.3)	0.528
No	229 (76.6)	129 (79.6)	358 (77.7)	
Involvement in drunkenness in the last 30 days				
Yes	122 (39.4)	188 (60.6)	172 (36.4)	0.086^*^
No	50 (30.9)	112 (69.1)	300 (63.6)	
Family income, minimal wages				
1-3	164 (83.2)	81 (77.9)	245 (81.4)	0.326
>3	33 (16.8)	23 (22.1)	56 (18.6)	
Age at first dose				
≤14	158 (62.7)	81 (66.9)	239 (64.1)	0.494
≥15	94 (37.3)	40 (33.1)	134 (35.9)	
Mother’s schooling level				
Incomplete Secondary Education or less	153 (61.2)	71 (53.8)	224 (58.6)	0.197^*^
Complete Secondary Education or further	97 (38.8)	61 (46.2)	158 (41.4)	
Father’s schooling level				
Incomplete Secondary Education or less	154 (66.7)	80 (70.2)	234 (67.8)	0.594
Complete Secondary Education or further	77 (33.3)	34 (29.8)	111 (32.2)	
Use of alcoholic beverage in the last 30 days				
No	159 (51.5)	91 (55.8)	250 (53.0)	0.419
Yes	150 (48.5)	72 (44.2)	222 (47.0)	
Use of alcoholic beverage during life, days				
0-9	215 (69.4)	101 (62.0)	316 (66.8)	0.076^*^
10-99	62 (20.0)	37 (22.7)	99 (20.9)	
≥100	33 (10.6)	25 (15.3)	58 (12.3)	

χ^2^ test. The total number may differ due to lost values. ^*p<0.20.^

Table 5 demonstrates binary logistic regression, and we can verify the association between the nonuse of male condom and the independent variables adjusted for gender of adolescents and youth. The Omnibus test presented with values of p<0.03, showing that the models were valid. In the Hosmer-Lemeshow test, which defined the explanatory power of the model, we found the value of 0.82 for females and 0.84 for males. There was an association between the involvement in drunkenness over the previous 30 days and female gender.

**Table 5:** Binary logistic regression analysis between non-use of male condom and the independent variables studied, adjusted by sex of adolescents and youth

Independent variables	Non-use of male condom			

	Gender female		Gender male	
	
	OR (95%CI)	p value	OR (95%CI)	p value
Involvement in drunkenness in the last 30 days				
Yes	2.19 (1.06-4.54)	0.034*	1.90 (0.98-3.68)	0.057
No	1		1	
Mother’s schooling level				
Incomplete Secondary Education or less	1.36 (0.75-2.45)	0.309	1.22 (0.70-2.12)	0.477
Complete Secondary Education or further	1		1	
Use of alcoholic beverage during life, days				
0-9	0.36 (0.12-1.01)	0.054	0.45 (0.18-1.08)	0.076
10-99	0.57 (0.20-1.65)	0.306	0.68 (0.26-1.77)	0.432
≥100	1		1	

The total number may differ due to lost values. *p<0.05. OR: *odds ratio*; 95%CI: 95% confidence interval.

## DISCUSSION

The epidemiological profile of adolescents and youth analyzed was marked by a majority aged between 14 and 16 years, single, with a predominance of the female gender, corroborating the findings of other national and international studies that showed similar profiles.^(1,6,16)^ A smaller portion reported not following any religion; the Christian religions were most prevalent, and most adolescents declared to be Catholic and were mulatto.

Thus, one can infer that the information relevant to the profile of adolescents and youth showed the characteristics of the Northeastern population, more specifically of the interior regions. On the national level, the contrast is evident between the Southern Region, with predominance of “Yellow” and “White” skin color, and the Northeast, in which “Black”, “Yellow”, and *“*Mulatto*”* represent the majority of the population.^(17)^

As to schooling level of parents, it was noted that the predominance was of those that had not concluded Secondary Education and had a monthly family income of up to two minimum wages, in agreement with data from other studies.^(12)^ For adolescents, low income represents a situation of vulnerability not only physiological, by means of exposure to diseases and poor nutrition, but also psychological, since it threatens confidence in their own future, and that of their communities and of their country.^(17)^

As to the sexual behavior of adolescents and youth, it was noted that the majority reported not having a sex life, and this behavior was greater in the younger age range. Several studies are in agreement with the data found, demonstrating a similar portion of adolescents with no sexual relations.^(1,6,12,14)^ The most prevalent age for sexual initiation was between 14 and 16 years and, although these data are in accordance with the mean age of literature, which is 15 years of age,^(18)^ these findings are concerning, since 37.9% reported having begun their sexual life before the age of 12; precociousness of sexual initiation makes these individuals sexually active for a longer period of time, and consequently, more susceptible to a greater number of sexual partners in life.^(19)^

In face of these findings, a study carried out in the United States with students from Secondary School in 16 states observed that in one third of the locations evaluated, sexual initiation before 11 years of age occurred for 8.1 to 10.3% of the sample.^(14)^However, some psychosocial aspects may contribute as a protective factor, delaying early sexual initiation, such as satisfaction in life, search for sensations, and religious support.^(16)^

As to the use of a contraceptive method during the last sexual encounter, it was noted, just as has been made evident by other authors,^(1,6,12,14)^ that most of the sample had used the male condom for the last sexual intercourse, and this was the method reported most frequently. It is believed that the positive behavior as to the use of the condom might be a result of campaigns and public policies that encourage and reinforce the practice of safe sex among adolescents.^(13)^ Measuring the use of contraceptive methods during the last sexual intercourse is an effective resource to identify contraceptive behavior in adolescence, considering the sporadic character of sexual relations, the dynamic of the sentimental relationships of this phase, and the alternating methods.^(20)^

Nevertheless, we should analyze these data with caution, since even with most of the sample having declared that they practice sexual prevention with the male condom, 34.4% reported not having used this method for the last sexual encounter, therefore continuing exposed to the occurrence of STD. Additionally, it was noted that 39% mentioned not using any method or other options, which would expose them to an undesired pregnancy.

A high percentage reported not having consumed alcohol at the last sexual encounter. Such a fact diverges from other studies that reveal the use of large quantities of alcoholic beverages before the last intercourse.^(12)^ Regarding this finding, it is believed that the findings of the present investigation could be elucidated considering the fact that the sample came from a region in the interior of the country, where the level of exposure and the influences related to the consumption of alcoholic beverages for this population group may have a different profile from that of large cities, which are locations where the majority of people within this context is developed.

Despite the greatest portion of the sample in this study reporting not having drunk an alcoholic beverage, among those that mentioned already having initiated the use of alcoholic beverages, a uniform distribution was noted among the age ranges, but the prevalence of the first dose was greater in adolescents aged 12 years or less, even though this practice is supported by law that prohibits the use of alcoholic beverages for this age range.^(21)^

In this way, such data should be seen with concern, since early initiation of alcohol use is directly associated with a greater chance of drunkenness episodes, both during adolescence and in adult life, as well as a greater probability of potential damage to the body.^(22)^ Additionally, prior studies demonstrated the existence of an association between early initiation of alcoholic beverage use with risky sexual behavior.^(23)^

An investigation developed by the Harvard Campus Alcohol Study, in 1999, showed that students who drank for the first time before the age of 13 years had twice the chance of practicing unplanned sex and 2.2 times greater chances of being involved in unprotected sex, when compared to students that initiated the use of alcoholic beverages after the age of 19 years.^(24)^

The most common pattern of consumption over the previous 30 days was that of one to nine doses, that is, most of the sample was a new user or an experimental user, while regarding involvement in drunkenness during this same period, the frequency found was that of five episodes. Drunkenness in this group is a behavior reported by several studies demonstrating that among adolescents who used alcoholic beverages, approximately half presented with at least one episode over the previous two weeks.^(6,12)^

In the association with the use of male condom, the variables included in the regression model were gender, involvement in drunkenness in the previous 30 days, mother’s level of schooling, and the use of alcoholic beverages in life. However, after the multinomial regression adjusted for gender of adolescents and youth, the only variable that remained unrelated to the use of male condom was the involvement in drunkenness in the previous 30 days.

It was noted that the girls who got involved in drunkenness in the previous 30 days presented with 2.19 more chances of not using the male condom. In this way, for the girls, the consumption of alcohol becomes a risk factor for unprotected sexual involvement, thus making them more vulnerable to STDs and to unplanned pregnancy. Data similar to the current study are found in other national and international studies, also reporting greater vulnerability for the female gender.^(6,12)^

Although our study made evident the association of nonuse of the male condom only for the female gender, a study that evaluated all 27 Brazilian states demonstrated the nonuse of the condom during the last sexual encounter was associated with some episode of drunkenness over the previous 30 days for both genders.^(6)^ Thus, regardless of the gender of the adolescent and youth, one should encourage dissemination of prevention and education measures regarding the consequences of the association of alcohol and unprotected sexual activity, but it is necessary that the focus of these policies be more persuasive and energetic for the female public.

The prevalence of unprotected sex may be determined by a high level of positive self-esteem and anxiety, associated by an insufficient level of knowledge, which are characteristics of this age range.^(1)^ In this sense, the anxiety of the girls facing their partners, associated with the lack of sufficient knowledge, may have contributed towards the association between drunkenness and no sexual protection.

Based on the results of this research, we can infer that the implications of this study are relevant, with data that characterize the local scenario. It is expected that the behavior patterns found may help in a better targeting of healthcare actions geared towards this public. A joint effort among the government, academia, and the civil society is needed to guarantee the regularity necessary for the conduction of studies about condoms, with the purpose of establishing a policy of monitoring of such indicators, as well as the unification of the measures taken, assuring a correct comparison between them.^(25)^

However, some limitations should be mentioned regarding the present study, such as the fact that the instrument used is based on self-reported answers, making it necessary to take into consideration the possibility of untrue answers.^(6)^ The research data came from a specific sample of a region in the interior of the state of Pernambuco, and it is not possible to infer that the conclusions found are applicable in other areas of Brazil or on a global level. Further, since it is a cross-sectional study, it is impossible to determine the causal effect of the risk behaviors evaluated.^(26)^

For future investigations, the development of studies with panel-type longitudinal designs is suggested, which would analyze adolescents and youth from different locations, assessing not only regional influences, but also between large cities and towns. Additionally, it is also important to evaluate if there are differences between the behavioral pattern of adolescents and youth with distinct socioeconomic levels.

## CONCLUSION

Most of the school-aged adolescents and youth reported having used the male condom in their last sexual encounter, and this is the most prevalent contraceptive method. Girls who had been involved in episodes of drunkenness in the previous 30 days showed higher chances of not having used the method in their last sexual relation. Finally, it is important to point out that, despite the association between not using the male condom and drunkenness having occurred only in the female gender, this finding still cannot be considered random, since statistical significance had been previously established and there was a plausible theoretical explanation for the fact.
